# Pathophysiology and Therapy of High-Altitude Sickness: Practical Approach in Emergency and Critical Care

**DOI:** 10.3390/jcm11143937

**Published:** 2022-07-06

**Authors:** Gabriele Savioli, Iride Francesca Ceresa, Giulia Gori, Federica Fumoso, Nicole Gri, Valentina Floris, Angelica Varesi, Ermelinda Martuscelli, Sara Marchisio, Yaroslava Longhitano, Giovanni Ricevuti, Ciro Esposito, Guido Caironi, Guido Giardini, Christian Zanza

**Affiliations:** 1 Department of Emergency Medicine and Surgery, IRCCS Fondazione Policlinico San Matteo, 27100 Pavia, Italy; gabrielesavioli@gmail.com; 2 Department of Emergency, Ospedale Civile, 27029 Vigevano, Italy; irideceresa@gmail.com; 3 Department of Internal Medicine, University of Pavia, 27100 Pavia, Italy; giulia.gori01@universitadipavia.it (G.G.); federica.fumoso01@universitadipavia.it (F.F.); 4 School of Medicine, University of Pavia, 27100 Pavia, Italy; nicole.gri01@universitadipavia.it (N.G.); valentina.floris01@universitadipavia.it (V.F.); angelica.varesi01@universitadipavia.it (A.V.); 5 Department of Emergency Medicine (ASL AL), San Giacomo Hospital, 15067 Novi Ligure, Italy; emartuscelli@asl.it (E.M.); saramarchisio@aslal.it (S.M.); lon.yaro@gmail.com (Y.L.); 6 Foundation “Ospedale Alba-Bra Onlus”, Department of Emergency Medicine, Anesthesia and Critical Care Medicine, Michele and Pietro Ferrero Hospital, 12060 Verduno, Italy; 7 Department of Drug Science, University of Pavia, 27100 Pavia, Italy; giovanni.ricevuti@unipv.it; 8 Nephrology and Dialysis Unit, ICS Maugeri, University of Pavia, 27100 Pavia, Italy; ciro.esposito@unipv.it; 9 Registered Nurse Supporting Prehospital Emergency Service ASST 118, 22100 Como, Italy; caironiguido@gmail.com; 10 Neurology and Stroke Unit, Ospedale Regionale “U.Parini”, 11100 Aosta, Italy; ggiardini@ausl.vda.it

**Keywords:** high mountain, acclimatization, acute mountain sickness, high-altitude cerebral edema, high-altitude pulmonary edema, emergency medicine, hostile environmental medicine

## Abstract

High altitude can be a hostile environment and a paradigm of how environmental factors can determine illness when human biological adaptability is exceeded. This paper aims to provide a comprehensive review of high-altitude sickness, including its epidemiology, pathophysiology, and treatments. The first section of our work defines high altitude and considers the mechanisms of adaptation to it and the associated risk factors for low adaptability. The second section discusses the main high-altitude diseases, highlighting how environmental factors can lead to the loss of homeostasis, compromising important vital functions. Early recognition of clinical symptoms is important for the establishment of the correct therapy. The third section focuses on high-altitude pulmonary edema, which is one of the main high-altitude diseases. With a deeper understanding of the pathogenesis of high-altitude diseases, as well as a reasoned approach to environmental or physical factors, we examine the main high-altitude diseases. Such an approach is critical for the effective treatment of patients in a hostile environment, or treatment in the emergency room after exposure to extreme physical or environmental factors.

## 1. High-Altitude Mountain Area as a Hostile Environment

### 1.1. Definition of High Altitude

High altitude is generally considered to be an altitude higher than 1500 m above sea level [1]. It is further classified as:

- High: 1500–3500 m. High-altitude sickness usually occurs during a rapid ascent above 2500 m (8202 ft). It is characterized by impaired physical performance and an increase in ventilation frequency, which is associated with a slight decrease in arterial oxygen saturation (SaO_2_) and an arterial partial pressure of oxygen (PaO_2_) of 55–75 mmHg;
- Very high: 3500–5500 m. Severe high-altitude illness most commonly occurs at this altitude, which requires a period of acclimatization n and where climbing, can be dangerous. SaO_2_ decreases to 75–85% and PaO_2_ to 40–60 mmHg; moreover, extreme hypoxia can occur during sleep or exercise;
- Extreme: 5500–8850 m. At these altitudes, there is progressive deterioration of physiological functions that quickly overwhelms the acclimatization process. Excessively rapid ascent almost always precipitates severe disease; with severe hypoxia and hypocapnia, SaO_2_ decreases to 58–75% and PaO_2_ to 28–40 mmHg.

### 1.2. Epidemiology

Across the world, people from low-altitude areas who undertake high-altitude journeys, primarily for work or tourism purposes, are at risk of high-altitude disease. With an increasing global demand for recreation and habitation at a high altitude, newcomers must familiarize themselves with the physical challenges and dangers associated with acute exposure.

High-altitude recreation has become increasingly popular, causing increased risks of AMS, which affects more than 25% of people who ascend to 3500 m (11,500 ft) and more than 50% of those who ascend above 6000 m (19,700 ft) [1,2,3]. In healthy people, AMS develops within a few hours at a high altitude. Its symptoms can include headache, anorexia, nausea, vomiting, dizziness, and fatigue [4,5]. In most cases, these symptoms resolve spontaneously after 18–36 h, without requiring a descent to a lower altitude. However, in less than 1% of AMS cases, the disease develops into life-threatening high-altitude cerebral edema, which is characterized by ataxia and an altered state of consciousness.

The likelihood of developing altitude sickness varies with each individual and ascent. Each ascent has variables, including ascent speed, altitude, atmospheric pressure, high-altitude sleep, time spent at a high altitude, exertion, temperature, pre-acclimatization, residence altitude, history of high-altitude illness, and history of pre-existing illnesses and drugs. The variables that determine the likelihood of high-altitude disease can be classified into environmental, behavioral, and intrinsic risk factors [5,6,7,8].

Due to the previously mentioned confounding variables, as well as differences in the study design and bias, the exact incidence of high-altitude disease has been difficult to determine [6,7]. Globally, the reported incidence varies significantly, from less than 10% to more than 90% for AMS and from less than 0.01% to 31% for HAPE and HACE.

The incidence and severity of high-altitude disease increase with the altitude and ascent rate; both factors affect the level of hypoxic stress [2,5,6]. The incidence rate of AMS in the general population, at around 2500 m (8202 ft) altitude, is reported to be 20–25%, increasing to 40–50% in trekkers and climbers near a 4000-m (13,123 ft) altitude [9,10,11,12,13,14,15]. When climbing to about 4000 m takes place over hours, rather than days, the incidence of AMS increases to more than 90% [16].

Similarly, the incidence rate of HACE in the general population at an altitude of about 2500 m (8202 ft) is reported to be less than 0.01%, but increases to 1–2% in trekkers, climbers, and soldiers near a 4000-m altitude [9,17,18,19,20].

The rate of HAPE incidence also increases with the altitude and ascent rate, ranging from 0.01–0.1% in the general population at 2500 m (8202 ft) to 2–6% in trekkers and mountaineers at 4000 m (13,123 ft) [10,12,20,21,22,23,24,25,26]. When a climb to 5500 m (18,044 ft) takes place over hours, rather than days, this incidence increases.

### 1.3. Environmental Stressors

High-altitude environmental conditions become increasingly hostile with further ascent, requiring greater behavioral and physiological adaptation to maintain vital functions.

Decreasing atmospheric oxygen pressure is the main environmental stress factor associated with high altitude; other environmental stressors are: low temperature and humidity and increased ultraviolet radiation.

High-altitude hypoxia is a direct result of the almost exponential fall in atmospheric pressure. The relative concentration of oxygen in the troposphere (the lowest atmospheric layer) is 20.93%. On ambient air, the partial pressure of oxygen (PO_2_) is obtained by multiplying 0.2093 by the atmospheric pressure corresponding to a given altitude. At sea level, PO_2_ is 0.2093 × 760 mmHg = 159.1 mmHg. On the top of Mount Everest, it is just 52.9 mmHg.

As a consequence of the falling atmospheric pressure, PO_2_ decreases in: ambient air, inspired air (PiO_2_), alveolar air (PAO_2_), and arterial blood (PaO_2_), whereas arterial oxygen saturation (SaO_2_) also decreases with progressive ascent [1,2,3]. Cellular processes are affected by the severity of hypoxia, which can vary at any altitude in relation to compensatory hyperventilation, lung function, and the affinity of hemoglobin for oxygen. It is important to note that respiratory depressants, vigorous exertion, hypothermia, and some pre-existing medical conditions aggravate hypoxemia [27].

Physiological adaptation to decreasing oxygen pressure in the high-altitude environment is known as acclimatization.

### 1.4. Physiological Response

#### 1.4.1. Acclimatization

Acclimatization is a complex and not yet fully understood mechanism to minimize hypoxemia and preserve cell function, despite reduced PO_2_. It involves roughly the whole body, with limits that vary between individuals, different ethnicities, and even animal species [27,28].

The symptoms of acute mountain sickness (AMS) (characterized by a varied set of symptoms, including headache, asthenia, dizziness) occur when people move to high altitudes from sea level, due to an inadequate acclimatization process. Such symptoms undergo rapid regression if the ascent is not progressed further; conversely, progression to HAPE or HACE may occur if the elevation process progresses despite symptoms, where HAPE and HACE, respectively, represent acute pulmonary edema and acute cerebral edema, the two major severe forms of altitude sickness.

It appears necessary to underline and add that, in some specific subpopulations, following the previous stay at sea level, HAPE re-entry pulmonary edema (HAPE) can occur [1]. A subgroup of HAPE, re-entry HAPE is a well-known life-threatening illness that has been recorded almost exclusively in North and South America. This phenomenon has not been reported in Sherpa or other people from Tibet in Nepal or India. It is most often seen in South Americans because of their poorer adaptation to the top altitude, which is probably due to the change in blood volume and the remodeling of the pulmonary arterioles with smooth muscle cell expansion, thus generating excessive pulmonary arteriole pressure upon the re-ascent to a high altitude. Regarding geographically isolated human populations, such as Tibetans and Andeans, who have a relatively different genetic basis, living for millennia in a very similar environment, they are under comparable selective pressure. The Andeans show a concentration of hemoglobin strongly dependent on altitude, in contrast to the Tibetans. The evolutionary pathway traveled by the Andeans increased their hemoglobin levels and selected a series of mutations in genes linked to the morphology of the cardiovascular system to make it more efficient in these subjects, resulting in very viscous blood (high level of hematocrit is a risk factor for the development of “chronic altitude sickness” CMS). The body exposed to high altitudes reacts with a form of hyperventilation known as HVR (hypoxic ventilatory response). Andean populations, if suddenly brought to high altitudes, can suffer an attenuated form of HVR, while Tibetans have a chronic form of HVR that allows them to breathe more air per unit of time. These two populations have adapted to the same environmental pressure in a genetically different way [27]. With sufficient time, most individuals can acclimatize to around 5500 m (18,045 ft). Above this level, progressive impairment prevails over compensatory adaptability [2,3].

#### 1.4.2. Respiratory System

An immediately efficient method to relieve hypoxemia is to increase ventilation [3]. Within minutes of climbing above 1500 m (4921 ft), both the tidal volume and frequency of breathing increase in proportion to the degree of hypoxemia detected by peripheral chemoreceptors (carotid bodies) [1,2,3]. This physiological response, known as the hypoxic ventilatory response (HVR), causes hyperventilation, which is responsible for a rapid decrease in the alveolar partial pressure of carbon dioxide (PCO_2_) and a proportional increase in alveolar PO_2_. Its effectiveness is partially diminished by consequent respiratory alkalosis, which inhibits the medullary respiratory center [2,29]. Interestingly, those who experience adaptation usually respond more strongly to future hypoxia after returning to normal oxygen status compared to non-adapted subjects. Signs of adaptation somehow make the body sensitive to hypoxia. These adaptations probably occur at the cellular level and are mediated by the hypoxia-inducing factor 1alpha (HIF-1α), an O2-sensitive transcription factor. The discovery of HIF1a and its role in cell perception and adaptation to the availability of O2 won William Kaelin Jr., Sir Peter Ratcliffe, and Gregg L. Semenza the 2019 Nobel Prize in Physiology or Medicine. Previous studies have shown that the peripheral chemoreceptor HIF1a is required for acclimatization. The respiratory center of the brainstem is sensitive to hypoxia and contributes to acclimatation, but it was previously unclear whether HIF1a was involved in the central nervous system’s contribution to acclimatization. HIF-1α is an important mediator of the cellular response to hypoxia. HIF-1α maintains oxygen homeostasis by inducing glycolysis, erythropoiesis, and angiogenesis [29]. However, ventilation continues to increase for approximately 7 days, despite persistent alkalosis in the blood and cerebrospinal fluid. This is partially explained by the increased renal excretion of bicarbonate that produces compensatory metabolic acidosis [1,2]. HVR is highly variable between individuals. It is sensitive to respiratory stimulants (e.g., acetazolamide, caffeine, coca, progesterone, and almitrine) and depressants (e.g., alcohol and sedatives). A vigorous HVR improves acclimatization, whereas a weak HVR can contribute to disease at a high altitude [1].

#### 1.4.3. Cardiovascular System

An initial increase in heart rate and pulmonary perfusion associated with the vasoconstriction and vasodilation of selective lung areas aim to optimize oxygenation in the lungs so as to supply well-oxygenated blood to the brain, heart, and others tissues [3]. Decreased arterial PaO_2_, detected by peripheral chemoreceptors, causes catecholamine secretion and activation of the sympathetic nervous system, thereby releasing epinephrine and norepinephrine. Although epinephrine activity is transient, norepinephrine stimulation persists for several days, which results in increased cardiac output, heart rate, peripheral vascular tone, blood pressure, and pulmonary perfusion. Subsequently, normalization of the heart rate and reduction in plasma volume (due to diuresis) decrease cardiac output. Finally, sensitivity to catecholamine decreases [1,2,3,30].

#### 1.4.4. Pulmonary Circulation

Alveolar hypoxia causes generalized pulmonary arterial vasoconstriction and a slight increase in pulmonary arterial pressure independent of the increase in cardiac output. This effect is known as the hypoxic pulmonary vasoconstrictor response (HPVR). It may initially improve the ventilation–perfusion ratio (V/Q) by redistributing blood flow to less-perfused areas of the lung [31,32]. Unfortunately, HPVR offers little adaptive advantage over its role in the pathogenesis of high-altitude pulmonary edema (HAPE) and in limiting application. The increase in pulmonary arterial pressure is rapid, inversely proportional to alveolar PO2, and highly variable among individuals. Exercise and cold increase pulmonary arterial pressure, whereas oxygen, descent, rest, and pulmonary vasodilator drugs reduce it [2,33,34,35,36,37,38,39,40,41,42,43,44]. Hypoxia is also a powerful factor that increases microvascular permeability. The significance of an increased capillary permeability in hypoxic edema formation is not clear. This can be accomplished through several mechanisms. In humans exposed to high altitude, permeability changes indicated by increased protein, inflammatory cytokines, and leukocytes in bronchoalveolar lavage appear to be a phenomenon secondary to inhomogeneous vasoconstriction. Here, we first recall the role of HIF-1α, a well-known factor that regulates cellular responses to hypoxic conditions. In particular, HIF-1α can increase microvascular permeability by inducing the high expression of matrix metalloproteinase 9. It is also necessary to remember that the upregulation of isthmin1 (ISM1) plays a fundamental role in increasing the permeability of alveolar epithelial cells.

ISM1 is regulated by HIF-1α, suggesting that HIF1α silencing inhibits the hypoxia-mediated upregulation of ISM1. The mechanism—overexpression of HIF1α—transcriptionally activates the expression of the ISM1 gene by directly binding to a conserved regulatory element upstream of the ism1 locus. Furthermore, hypoxia and impaired mitochondrial function may cause apoptotic cell death. Elevated necrosis is accompanied by significant extracellular HMGB1 release, in contrast to the consequences of apoptosis. This leads to inflammatory activation and an increase in capillary permeability [2,33,34,35,36,37,38,39,40,41,42,43,44].

#### 1.4.5. Cerebral Circulation

Cerebral oxygen consumption accounts for approximately 20% of the body’s total resting oxygen consumption. There is a delicate self-regulated system for cerebral circulation that is highly sensitive to hypoxia. Blood flow quickly increases in response to a hypoxic stimulus. There is a slight increase in cerebral flow, proportional to the degree of hypoxemia, which is more evident when PaO_2_ falls below 60 mmHg [1,2,31,45].

#### 1.4.6. Hematopoietic Blood System

Hematological changes increase arterial oxygenation. The blood’s oxygen content rapidly increases during the first few days at a high altitude because of hemoconcentration, due to diuresis and the contraction of plasma volume, but also due to the hypoxia-induced secretion of erythropoietin that begins hours after ascent to a high altitude. It stimulates the production of red blood cells in the bone marrow, which takes weeks, due to the increase in red blood cell mass. This increase in erythropoietin offers little or no short-term benefit. In the long term, poly-erythrocythemia plays a role in the development of chronic mountain sickness and in the limitation of the ability to exercise at a high altitude [2,46,47]. The affinity of hemoglobin for oxygen affects how oxygen is taken up within pulmonary capillaries and how it is released into tissue capillaries, as described by the oxygen–hemoglobin dissociation curve. At high altitudes, persistent respiratory alkalosis and increased concentrations of 2,3-diphosphoglyceric acid compete to influence the curve position. At modest altitudes, the result do not change. At very high and extreme altitudes, a sharp shift to the left of the curve supplies more oxygen to the lungs, preserving arterial oxygen levels [1,2,3,47,48,49].

The tendency toward inadequate acclimatization and susceptibility to high-altitude disease significantly varies between individuals and populations. This variability has a genetic basis; it depends on intrinsic anatomical and physiological differences.

Environmental conditions, behavioral factors (such as ascent rate and use of ventilatory depressants), viral respiratory infections, and the presence of certain pre-existing diseases can potentially aggravate hypoxemia [31,50,51,52].

Various risk factors can compromise acclimatization.

### 1.5. Risk Factors

#### 1.5.1. History of Previous High-Altitude Illness

A previous history of high-altitude illness indicates an individual’s susceptibility; thus, patient history is the most valuable predictor of illneses. For example, the incidence of HAPE after climbing to 4559 m (14,957 ft) increases from 10% to 60% in mountaineers with a radiographically documented history of HAPE [21,53]. Although there is no simple method for predicting the risk of recurrence, high-altitude diseases generally recur in susceptible individuals during subsequent exposure to the same altitude at a similar rate of ascent. On the contrary, people who tolerate high altitude without symptoms usually experience no symptoms during subsequent visits to the same altitude at a similar rate of ascent [6,54]. Regardless of how acclimatization limits are exceeded, high-altitude hypoxemia results. Hypobaric hypoxia and hypoxemia are the pathogenetic precursors of high-altitude disease [31,55,56].

#### 1.5.2. Gender and Age

Most epidemiological studies of high-altitude disease are limited to relatively homogeneous human populations, making it difficult to accurately understand the influence of age and gender. Acute mountain sickness (AMS) is reported equally in both genders. Reported cases of HAPE and HACE are predominantly males, but this may be related to the study population, rather that reflecting prevalence among genders. The risk of AMS is 2.06 times (95% confidence interval [CI], 1.15–3.72) lower for people older than 50 years [7,17,54,57,58,59]. AMS is less common in adults older than 50 years than in children and younger adults. HAPE appears to occur more frequently and with greater severity in children and young adults and without gender preferences in these age groups [1,2,10,18,22,23,57,60].

A role could also be played by menopause. In fact, it has been highlighted that postmenopausal woman have adaptive ventilatory responses that are less pronounced or absent compared to men of the same age, and that training can reduce or cancel this effect. [9].

#### 1.5.3. Fitness and Physical Exercise

Fitness is not protective against high-altitude disease, nor does it improve the ability to acclimatize. Vigorous effort is harmful when first arriving at a high altitude [2,3,4,9]. However, fitness is associated with an increased ability to perform vigorous activity at a high altitude.

#### 1.5.4. Pre-Acclimatization

Pre-acclimatization offers some protection against high-altitude diseases. A slow ascent rate and living at an altitude higher than 900 m (2953 ft) are associated with lower incidence and severity of disease during ascent. However, this protection is not complete; high-altitude sickness can still occur if the ascent is too rapid, or at an extreme altitude [2,5,6,9,17].

#### 1.5.5. Drugs and Poisoning

Alcohol, barbiturates, opiates, and other ventilatory depressants can change sleep patterns, reduce ventilation, and exacerbate high-altitude hypoxemia. Despite the lack of evidence that these substances increase the incidence or severity of high-altitude disease, pathophysiological data recommends the avoidance of ventilation depressants [1,50].

#### 1.5.6. Pre-Existing Diseases

Although many pre-existing diseases can be exacerbated by high altitude, some increase susceptibility to high-altitude disease. The risk of HAPE is increased by congenital abnormalities of cardio-pulmonary circulation, such as unilateral absence of the pulmonary artery, pulmonary arterial hypertension, coagulopathies, bleeding disorders, previous spontaneous or traumatic hemorrhages, and congenital heart defects causing chronic secondary pulmonary hypertension [21,61,62,63,64,65,66,67,68,69,70,71].

#### 1.5.7. Cold Weather

Cold constitutes an additional physiological stress that elevates pulmonary arterial pressure. It is a risk factor for HAPE [3].

### 1.6. High-Altitude Disease Classification

High-altitude disease develops when stressful environmental factors exceed the adaptability of the organism, especially in the presence of other risk factors. It is directly caused by morbid conditions at a high altitude; pre-existing medical problems are aggravated by altitude. The term high-altitude sickness is specific for three pathologies, characterized by cerebral and pulmonary alterations arising from acute exposure to high altitude and hypobaric hypoxia:

- Acute mountain sickness;
- High-altitude cerebral edema;
- High-altitude pulmonary edema.

Despite the fact that high-altitude disease is preventable, AMS, HACE, and HAPE are common consequences of rapid ascent to high altitude.

## 2. Acute Mountain Sickness and High-Altitude Cerebral Edema

### 2.1. Acute Mountain Sickness: Definition, Diagnosis, and Clinical Considerations

AMS (Acute Mountain Sickness) is a self-limiting disease, with its main threat being the potential progression into HACE (high-altitude cerebral edema) [1].

The most widely used consensus document (with the greatest degree of reliability, reproducibility, and accuracy) is the Lake Louise Questionnaire Score (Table 1). It is an AMS scoring system, first published in 1991, which is widely used today to assess the severity of illness. The Lake Louise AMS score for an individual is the sum of the score for the four symptoms (headache, nausea/vomiting, fatigue, and dizziness/light-headedness). For a positive AMS definition, it is mandatory to have a headache score of at least one point, and a total score of at least three points. Recent studies have shown that disturbed sleep at altitude, one of the five symptoms scored for AMS, is more likely due to altitude hypoxia per se, and is not closely related to AMS [72]. The onset after 3 days and without headache may be indicative of other diagnoses.

Based on LLQS score, AMS is classified as follows:

- Mild: 3–5 points
- Moderate: 6–9 points
- Severe: 10–12 points

Although symptoms may develop within 6 h, it is recommended to calculate the AMS score only after 6 h to exclude travel symptoms and acute hypoxia.

AMS symptoms usually develop at altitudes above 2500 m (8202 ft), but sometimes, they commence at 2000 m. They may arise 6–10 h after ascent, but sometimes as soon as 1 h after ascent. Another diagnosis must be suspected in the presence of onset of symptoms after 3 days of stay at the same altitude, the absence of headache, or the non-resolution of symptoms following the descent or the administration of oxygen.

Other diagnoses that need to be considered in the early assessment of possible AMS or HACE include subarachnoid hemorrhage, intracranial mass, migraine, dehydration, exhaustion, exposure to carbon monoxide, and substance abuse [1,57].

The results of noninvasive pulse oximetry are inversely related to the severity of AMS, and may detect extreme hypoxemia in severe AMS [60]. However, correlation between symptoms and pulse oximetry is low; oxygen saturation percentage is not required for diagnosis.

High-altitude cerebral edema (HACE) is a neurological syndrome that is considered to be the last phase of AMS. It occurs within hours or days in patients who have already developed AMS or HAPE (high altitude pulmonary edema) and is potentially fatal.

The onset of HACE usually occurs 3–5 days after ascending to 2750 m (9022 ft). The incidence of HACE is greater at higher altitudes, and its onset is usually abrupt.

The diagnosis of HACE is based on clinical signs. The main symptoms (Table 2) are alterations of behavior and consciousness, such as clouding of the senses, lethargy (until coma), and ataxia [4]. The absence of coordination between the trunk and lower or upper limbs (truncal ataxia) is a typical sign. Papilledema, retinal hemorrhages, cranial nerve palsy, abnormal reflexes, and (rarely) focal neurological deficits may be present. Death is usually caused by cerebral herniation [1,2,57].

### 2.2. Pathophysiology

#### 2.2.1. Overall

AMS and HACE represent a pathophysiological continuum.

The manifestations of HACE may be considered as a clinical evolution of AMS; both involve neurological dysfunction. Because the boundaries between AMS and HACE have not been defined, some authors have proposed the term AMS/HACE, denoting the underlying intrinsic pathological connection. At one end of the continuum are mild AMS symptoms; at the other end are the severe and potentially fatal signs of HACE.

#### 2.2.2. Hypoxia as a Primum Movens: Bio Humoral Response and Fluid Mechanics

While ascending to a high altitude, susceptible patients may develop relative hypoventilation and hypoxia, triggering multiple pathological responses that lead to the onset of disease. However, factors other than hypoxia are needed to fully explain disease onset, as has been demonstrated in several studies. Greater capillary permeability induced by the complex interaction between fluid mechanics (regional hyperperfusion and high hydrostatic pressure) and biohumoral response (mediators of inflammation release), together with the impairment of compensatory mechanisms (e.g., reduced capability to amortize intracranial volume variations and the impairment of alveolar fluid’s transepithelial transport), lead to cerebral vasogenic edema (AMS/HACE) and pulmonary hydrostatic edema (HAPE) [21,50,73].

#### 2.2.3. Hypoxia and the Neurotransmitter Hypothesis

Accurate measurements of cerebral metabolism and oxygen supply are not available for patients with high-altitude illness. Studies of sheep have demonstrated that if acclimatization occurs correctly at moderate altitudes, metabolism is properly maintained. This is indicated by normal oxygen pressure and the presence of metabolites in venous cerebral flux [58].

The measurements in the sheep model show that cerebral edema in AMS/HACE may occur in the absence of cerebral hypoxia. Indeed, sheep have exhibited AMS/HACE symptoms, despite normal oxygen intake (when oxygen was artificially augmented, as at sea level). Oxygen fraction and global oxygen consumption were stable and within the normal range. HACE may be explained by neurotransmitter impairment, influenced by hypoxia. Hypoxia levels alone are not sufficient to affect global metabolism and energetic balance.

The neurotransmitters most involved are serotonin, dopamine, and acetylcholine, which are all sensitive to low oxygen levels. This has been demonstrated in animals at a high altitude, where, for example, serotonin synthesis is decreased [50].

A recent study has proved that dopamine synthesis is improved during hypoxia, suggesting that changes in post-synaptic receptors and signal transduction might be the causes of impaired dopaminergic function [3]. Moreover, some authors indicated that dopaminergic drugs (such as amphetamine), or a diet rich in tyrosine, may improve cognitive function and well-being at a high altitude [2,3,21,48,50,58,73,74]

Acetylcholine depletion would seem to be the cause of fatigue, which is common at a high altitudes [21,50,73].

Neurotransmitter impairment may explain cognitive deficit and mood changes.

#### 2.2.4. Biohumoral Response

Studies of sheep have demonstrated the existence of other factors responsible for the increase in capillary permeability, aside from vasodilation. These factors include hypoxia-induced chemical mediators, such as histamine, arachidonic acid, reactive oxygen species, and nitric oxide.

Nitric oxide is essential for the proper functioning of the blood–brain barrier [14,22]. Some form of inducible nitric oxide synthase may be induced by hypoxia. The nitric oxide produced by nitric oxide synthase might cause cerebral edema [75], increasing blood–brain barrier permeability through interactions with inflammatory cytokines.

#### 2.2.5. Vasogenic Edema

Vasogenic edema is an extracellular edema that affects mainly white matter through the leakage of fluid from the capillaries. It is the main cause of HACE [76]. A magnetic resonance imaging study of nine men affected by HACE (eight of whom also had HAPE) demonstrated hyperintense T2 and FLAIR signals at the level of the corpus callosum in seven cases, but without restricted diffusion, which verified that the cause of edema was increased capillary permeability, not a cytotoxic process.

Gray matter is formed by tight, tangled cellular structures, whereas white matter has a tidy network of extracellular meshes; it is less thick and offers less resistance to the formation of edema. This is the reason vasogenic edema preferentially spreads through the white matter. Klatzo has compared vasogenic edema to an overflowing river. In contrast to cytotoxic edema, permanent cerebral damage does not occur if treatment is initiated before the occurrence of ischemia [19].

The pathophysiological mechanism of AMS/HACE is proven to be the basis of vasogenic edema by the following evidence: disease course (from the symptoms at onset to resolution), several experiments on animals [22], clinical responses to corticosteroids, the absence of neurological sequelae, and hypoxia-induced increase in endothelial permeability in vitro.

#### 2.2.6. Anatomical Variability

An anatomical explanation for AMS was proposed by a neurologist named Ross in 1985 [11]. He proposed that patients with reduced capacity to host cerebrospinal fluid would be more susceptible to a slight increase in cerebral volume and would develop AMS more easily. Hackett renamed this hypothesis “Tight Fit”. It might explain both individual susceptibility and poor correlation with the physiological variables examined to date.

### 2.3. Prevention

It is possible to predict who will develop high-altitude disease using the Richalet hypoxia sensitivity test, indicated for those who have never been at a high altitude and who face a journey to a high altitude without the possibility of acclimatization (e.g., a business trip to Bolivia or tourism in Chile at 5000 m); the positive predictive value of this test is 79% [YY]. AMS has no predictors. The most reliable risk indicator may be a positive anamnesis of a previous episode.

Acclimatization is the most important measure for decreasing the risk of AMS/HACE/HAPE. The speed of ascent should be around 600 m/day, and climbers should rest for 1 day for every 600–1200 m of ascent; this only applies to those who are at low risk of developing complications and with no previous episodes of altitude sickness.

Early symptoms should be quickly recognized, especially regarding HACE. All climbers should strictly follow prevention guidelines.

Prophylaxis with acetazolamide (125–250 mg orally, twice daily) may be useful for people with a history of high-altitude disease [1].

### 2.4. Future Progress

There is a solid consensus that work is required in the following areas:

- Research to better understand the pathophysiology of AMS/HACE/HAPE, for the development of new therapeutic strategies;
- An AMS score should be established for assessing clinical and functional impacts;
- Training in AMS scoring should be provided to physicians and lay people;
- The impact of insomnia on general well-being at a high altitude should be assessed;
- Research is needed into the pathophysiological differences between typical AMS and non-headache AMS.

## 3. High-Altitude Pulmonary Edema

### 3.1. Definition, Diagnosis, and Clinical Considerations

According to the Lake Louise questionnaire (Table 3), HAPE can be diagnosed in the presence of at least two of the following symptoms: dyspnea, cough, asthenia, reduced physical performance, chest tightness, and chest congestion and at least two of the following: crackles, whistles, tachypnea, or tachycardia [4]. Early pulmonary edema may manifest only as reduced physical performance and dry cough, which the patient may minimize or ignore.

Progression to a more severe clinical status may occur within a few hours or days. The onset of severe forms is characterized by pinkish expectoration, severe dyspnea, and even death [58]. HAPE is the major cause of death among high-altitude syndromes. It rarely occurs at altitudes lower than 2440 m (8000 ft) [57]. The rate of progression is accelerated by exposure to cold, vigorous exertion, and climbing.

HAPE begins within 1 to 3 days after reaching a new altitude, and rarely after 4 days [5]. After the fourth day, HAPE is unlikely; other diagnoses should be considered, such as pneumonia, cardiogenic pulmonary edema, and spontaneous pneumothorax.

The diagnosis of HAPE is made by clinical suspicion, based on the symptoms and on the detection of a reduced oxygen saturation of the peripheral blood through a pulse oximeter; therefore, no other medical, laboratory, or imaging tests appear necessary.

Thoracic X-ray shows characteristic pulmonary thickening but not cardiomegaly or Kerley B lines. Thoracic X-ray findings include unilateral or bilateral, central or peripheral, homogenous or patchy fluffy opacities, predominantly in the dependent zones of the lungs.

Plainly, HAPE is a hydrostatic edema [35]. The relevant literature is unclear as to how rapid ascent, very high altitude, and severe exertion predispose the onset of HAPE. Clearly, some are predisposed; pulmonary arterial pressure rapidly increases in response to alveolar hypoxia—HPVR [77]. HPVR is caused by relative hypoventilation and alveolar hypoxia, together with increased sympathetic activity and lower endogenous nitric oxide production [21]. Pulmonary arterial vasoconstriction is uneven due to anatomical features. This leads to uneven regional perfusion, which is visible as irregular lung thickening, and is associated with alveolar–capillary barrier impairment

Mechanical alterations alone may not explain persistent edema in HAPE. It is possible that inflammatory mediators play a role; their activation would seem to be secondary to the mechanical changes caused by hydrostatic overload [68,78,79]. Recent data suggest that impairment in transepithelial sodium transport in type 2 alveolar cells may confer a predisposition to edema overload and thus, to the onset of HAPE [73,80,81].

### 3.2. Pathophysiology

The exact cause of HAPE remains unknown. Patients with HAPE have increased pulmonary arterial pressure and normal left atrial pressure [82,83,84,85,86,87], increased pulmonary vascular responsiveness to hypoxia, and decreased pulmonary arterial pressure. It is improved by intervention [88,89,90,91,92,93,94]. These observations are consistent with the hypothesis that HAPE is caused by pulmonary capillary stress disorders associated with heterogeneous hypoxic vasoconstriction and overflow [95].

However, bronchoalveolar lavage fluid has been shown to be rich in high molecular weight proteins, cells, and inflammatory markers in HAPE patients [96], suggesting increased capillary permeability as a major event. In addition, Maggiorini et al. found that patients with early HAPE had pulmonary capillary pressure above 19 mm Hg and a normal pulmonary leakage index. This suggests that HAPE is initially hydrostatic pulmonary edema [35].

In HAPES mountaineers (sensitive to high-altitude pulmonary edema), vasoconstriction was also recorded at the systemic level after exposure to hypoxia (armpit blood flow), as opposed to non-HAPES patients. This finding was due to impaired vascular endothelial function resulting from the reduced bioavailability of NO [41]. A decrease in exhaled NO was also seen in HAPES patients and HAPE patients exposed to hypoxia [42,43]. Based on causality, it remains unclear whether low NO bioavailability is due to impaired biochemical metabolic pathways or, conversely, that it merely represents a functional response to counteract the formation of edema.

### 3.3. Prevention

Patients who have previously been affected by HAPE should consider pharmacological prophylaxis when climbing to a high altitude. A gradual ascent (1 day of rest every 600–1200 m) at a slow speed (maximum 600 m/day) enables appropriate acclimatization and decreases the risk of HAPE relapse. This is particularly true for those who have never suffered from AMS or its complications; on the contrary, those who have already suffered from AMS/HACE or HAPE must climb much slower, even 300 m/day. Patient should avoid severe exertion for the first 3 days. Above all, if the patient is fatigued, this might be a subclinical sign of HAPE.

All climbers should avoid taking ventilation depressors, such as alcohol. Extended release nifedipine is effective in lowering pulmonary arterial pressure and preventing HAPE. Salmeterol improves symptoms in climbers who have been previously affected by HAPE [73].

Acetazolamide, at a dose of 125 mg twice daily, is used to facilitate acclimatization and reduce hypoxemia in patients who have previously been affected by HAPE. This treatment must be initiated 1 to 2 days before climbing and continued for 2 days after having reached the maximum altitude. Physical conditioning does not prevent HAPE [1,3,80].

HAPE can be lethal. If it is not recognized promptly, it can quickly progress to severe encephalopathy and coma.

## 4. Therapy

### 4.1. Acute Mountain Sickness and High-Altitude Cerebral Edema

The treatment of the mild-moderate form of AMS (rest, NSAIDs, antiemetics) is different from that of the severe form and of HACE (imperative descent, hyperbaric caisson, oxygen, steroids). Hyperventilation can momentarily improve symptoms. In severe cases, therapy should aim to reduce intracerebral volume and intracranial pressure (ICP). This can be achieved by (1) administering oxygen therapy with increased inspired oxygen fraction; (2) bringing the patient to a lower altitude; (3) using a hyperbaric chamber, which immediately decreases cerebral blood flow (CBF) and, thus, ICP; and (4) using drugs to halt cerebral edema formation.

New therapies will be developed and will become available when a full understanding of the pathophysiological mechanisms is achieved.

We include the following drugs in our study (Table 4):

- *Carbonic anhydrase inhibitors*: (acetazolamide and methazolamide). Carbonic anhydrase inhibitors are diuretics that act on the proximal tubule to cause metabolic acidosis and loss of carbonic acid. Metabolic acidosis leads to hyperventilation, which improves ventilation in response to high-altitude hypoxic stimuli [97]. Acetazolamide may also provoke pulmonary vasodilatation, not correlated with carbonic anhydrase inhibition [98], which improves oxygenation, increases ventilation, halts cerebrospinal fluid formation, and forces diuresis. This drug can be useful as a prophylactic therapy. However, it is not able to reduce CBF or ICP.
- *Corticosteroids*: (dexamethasone and medroxyprogesterone). Dexamethasone reduces hypoxia-induced endothelial impairment [99]. The possible mechanism involves the inhibition of angiogenesis and lipid hyper oxidation, stabilization of mast cell membranes, and influence on the production of inducible nitric oxide synthase. Medroxyprogesterone acts as a ventilation stimulant [100]. In vitro studies demonstrate that corticosteroids decrease hypoxia-induced endothelial permeability in the brain [101].
- *Non-steroidal anti-inflammatory drugs*: (ibuprofen, paracetamol, and aspirin). Prostaglandins contribute to an increase in cerebral vascular permeability in AMS. Therefore, prostaglandin synthetase inhibitors may be used to manage this mechanism [67].
- *Selective 5-hydroxytriptine receptor agonists*: (sumatriptan). These selective cerebral vasoconstrictors are used to reduce cerebral vascular permeability [5,102].
- *Anticonvulsant drugs*: (gabapentin). Gabapentin has analgesic properties [103,104].

Finally, where a real descent is not practicable, it is possible to resort to hyperbaric therapy (chambers, manual pneumatic pump, portable hyperbaric bag). Hyperbaric therapy simulates a descent in altitude. It improves symptoms and increases arterial oxygenation. This is used as a temporary treatment [67].

### 4.2. High-Altitude Pulmonary Edema

Appropriate therapy depends on disease severity, available treatment options, proximity to medical care facilities, and the altitude where symptoms occurred. Most patients benefit from descent and treatment with hyperbaric or oxygen therapy, all leading to increased PiO_2_, which immediately increases arterial oxygenation, protecting the brain, and reducing pulmonary arterial pressure, heart effort, respiratory rate, and dyspnea.

Even if treatment immediately improves a patient’s condition, complete recovery usually takes days. Patients must remain warm and rested. Cold, stress, and exertion increase pulmonary arterial pressure.

Oxygen therapy must be immediately delivered. When oxygen therapy is not available, it is useful to administer acetazolamide (250 mg, twice daily) when descent is delayed, even if it may increase dyspnea. Dexamethasone does not improve the clinical outlook in HAPE [105].

From the pathophysiological point of view, beta-agonists (salmeterol, 125 mcg, inhaled every 12 h, or salbutamol, four to six times daily) may be used, although it is not known whether they provide benefit when used in addition to oxygen therapy.

If a patient wishes to remain at the altitude and a hospital is available, the usual practice is a 2–3-day hospital stay, or ambulatory oxygen therapy. The patient should not fly while oxygen therapy is required.

Hyperbaric therapy may be available outside the hospital (e.g., in resort areas). Even though it is an effective therapeutic method, it is more expensive than oxygen supplementation, and there is no evidence that it is superior.

The patient may return to his/her activities after symptoms have resolved and oxygen saturation is greater than 90% [3,31,57,80,106].

To summarize, pharmacological therapy consists of the following:

- *Calcium channel blockers*: (nifedipine). Calcium channel blockers reduce pulmonary vascular resistance.
- *Nitric oxide*: Nitric oxide is an endothelial vasodilating factor, produced during hypoxia. It reduces pulmonary vasoconstriction [94,107,108,109].
- *Non-selective phosphodiesterase inhibitors*: (theophylline, aminophylline). The antioxidant effects of non-selective phosphodiesterase inhibitors may reduce periodic breathing, lung and brain microvascular permeability, and pulmonary arterial pressure [110,111].
- *Positive pressure on respiratory tract*: Breathing against positive pressure improves arterial oxygen saturation [72,112,113,114,115].

## 5. Conclusions

The physiological limitations of the human species to adapt to acute hypobaric hypoxia, combined with the desire to visit high-altitude destinations in a cost-effective and rapid manner, ensure that these diseases will not disappear. As the global popularity of recreating and living at high altitudes continues to increase, people who work with or advise those traveling to high altitudes need to be familiar with early symptom recognition, prompt and appropriate therapy, and adequate preventive measures to reduce the morbidity of high-altitude disease.

## Figures and Tables

**Table 1:** Lake Louise Questionnaire Score for diagnosis of AMS.

Symptoms	Score
Headache	0—None at all 1—A mild headache 2—Moderate headache 3—Severe headache, incapacitating
Gastrointestinal symptoms	0—Good appetite 1—Poor appetite or nausea 2—Moderate nausea or vomiting 3—Severe nausea and vomiting, incapacitating
Fatigue and/or weakness	0—Not tired or weak 1—Mild fatigue/weakness 2—Moderate fatigue/weakness 3—Severe fatigue/weakness, incapacitating
Dizziness/light-headedness	0—No dizziness/light-headedness 1—Mild dizziness/light-headedness 2—Moderate dizziness/light-headedness 3—Severe dizziness/light-headedness, incapacitating

**Table 2:** Symptoms of HACE.

Main Symptoms	Typical Symptoms	Rare Symptoms
Clouding of the senses, lethargy (until coma), ataxia.	Absence of coordination between the trunk and lower or upper limbs (truncal ataxia).	Papilledema, retinal hemorrhages, cranial nerve palsy, abnormal reflexes, focal neurological deficits.

**Table 3:** Diagnostic criteria for HAPE.

**A.** **At least two of these symptoms:**	Dyspnea Cough Asthenia Reduced physical performance Chest tightness Congestion
**B.** **At least two of these symptoms:**	Crackles Whistles Tachypnea Tachycardia

**Table 4:** Main drugs used for treatment of HACE.

Class of Drug	Example	Mechanism
*Carbonic anhydrase inhibitors*	Acetazolamide, methazolamide	Diuretic effect → metabolic acidosis and loss of carbonic acid → hyperventilation → compensates metabolic acidosis with respiratory alkalosis.
*Corticosteroids*	Dexamethasone, medroxyprogesterone	Inhibition of angiogenesis, lipid hyper oxidation, stabilization of mast cell membranes, influence on the production of inducible nitric oxide synthase → reduces hypoxia-induced endothelial impairment.
*Non* *-* *steroidal anti-inflammatory drugs*	Ibuprofen, paracetamol, aspirin	Contribute to an increase in cerebral vascular permeability.
*Selective 5-hydroxytriptine receptor agonists*	Sumatriptan	Selective cerebral vasoconstriction → reduce cerebral vascular permeability.
*Anticonvulsant drugs*	Gabapentin	Analgesic properties.
*Hyperbaric therapy*	Chambers, manual pneumatic pump, portable hyperbaric bag	Simulation of a descent in altitude → improve symptoms and increases arterial oxygenation.
